# Evaluation of the Synthesized Novel Iridium (III) Complexes Against HeLa Cell Lines Through *In-Silico, In-Vitro* and DNA Nicking

**DOI:** 10.31557/APJCP.2021.22.2.447

**Published:** 2021-02

**Authors:** G. Sathya Priyadarshini, Aathi Muthusankar, Ramesh Subramani, Selvi Gopal

**Affiliations:** 1 *Department of Chemistry, PSGR Krishnammal College for Women, Peelamedu, Coimbatore, India. *; 2 *Membrane Protein Biology Group, International Centre for Genetic Engineering and Biotechnology, New Delhi 110067, India. *; 3 *Department of Food Processing Technology & Management, PSGR Krishnammal College for Women, Peelamedu, Coimbatore, India. *

**Keywords:** Iridium complexes, quinoline schiff base, molecular docking, DNA nicking

## Abstract

Globally, the pharmaceutical industry is continuously driven in search of new anticancer drugs due to increasing rate of cancer patients. Clinical trials of Cisplatin has been explored, however, usage of Cisplatin as a drug is limited due to its various side effects, hence, alternative to platinum based complex drugs and its analogues are needed. Iridium complexes have been attracted widespread interests by virtue of their pharmacological and photo-physical properties; however the less number of complexes was reported in the literature. In this article, a new series of novel Iridium (III) complexes were synthesized using substituted quinoline Schiff Base (SB) ligands and characterized by spectroscopic techniques. The *in- vitro* cyto-toxicity assay showed that the Iridium (III) complex activity is equal to standard Cisplatin. In addition, computational docking studies have shown that the prominent binding sites for synthesized complexes against HeLa cell lines, which is comparable with standard Cisplatin drugs and other Ruthenium complexes.

## Introduction

Transition metal based inorganic complex is playing a predominant role in the field of medicinal chemistry (Ndagi et al., 2017). The Pharmaceutical industry is constantly searching for novel anticancer drugs as urged by WHO due to the alarming rate of cancer patients (Freddie et al., 2020). It is estimated by WHO that more than 30 million people globally are in the need of anti-cancer drugs in the coming years. Clinical trials of Cisplatin (CP), platinum group of complexes were studied to treat cervical, ovarian, testicular, head and neck, breast, bladder, stomach, prostate and lung cancers (Bai et al., 2020; Mukhopadhyay et al., 2015). The usage of CP as a drug is limited due to its various side effects such as neutropenia, lymphedema, deep vein thrombosis, chemo brain, hair loss and vomiting [https://www.cdc.gov/cancer/survivors/patients/side-effects-of-treatment]. As a result, researchers are focused on designing of novel anticancer drug that can specifically identify tumor targets to overcome the drawbacks of chemotherapeutic agents. Hence, alternative platinum based complex drugs and its analogues viz. Carboplatin, Oxaliplatin, Satraplatin, Aroplatin, Enloplatin, Zeniplatin, Sebriplatin, Miboplatin, Picoplatin, and Iproplatin were investigated (Florea et al., 2011; Monneret, 2011; Wheate et al., 2020). 

Dose related factors of platinum drugs have led to the exploration of other elements of the same series of metals. For instance, Ruthenium (Ru) and Iridium (Ir) complexes have gained significant attention for last few years due to its anticancer properties with low side effects (Sudding et al., 2014). However, Ir complexes containing NNO donors are explored to a lesser extent which can act as a better anticancer drug due to their kinetic aspects, water solubility and binding ability with DNA (Adhikari et al., 2017; Sliwinska-hill et al., 2013; Adhikari et al., 2016; Mandal et al., 2014). To make such effective anti-cancer complex drug with transition metal, ligand design is challenging to maintain its physicochemical properties such as fluorescence, photo activity and cytotoxicity. Schiff Base (SB) is being the better benign for complexation with metals as many studies were reported in the literature. SB complexes tethered with heterocyclic moieties like 4-aminoantipyrine, pyrazole, 1,2,4-triazoles, benzoxazole, triazines, and coumarins have received remarkable interest as broad-spectrum due to their wide range of properties such as antibacterial, antifungal and antiviral agents (Chow et al., 2013). For example, highly hydrophobic Ir (III) complexes containing both cyclopentadienyl group and a C,N-chelating ligand have been validated their significant cytotoxic activity towards A2780 human ovarian cancer cells (Lu et al., 2015). Luminescent cyclometalated Ir (III) complexes were reported for their strong selectivity for cancer cells over normal cells and as specific inhibitors for protein-protein inhibition (Śliwińska-Hill et al., 2013; Xu et al., 2020). 

The main reason for choosing the Iridium is due to the inertness of Iridium complexes with 5d^6^ low spin configuration which inhibits the kinase enzymes (significant in angiogenesis in tumor progression). In this article, a series of new Iridium (III) complexes bearing a salicylidene hydrazino and thiosemicarbazino ligand and different types of substituent in quinoline (N^N) ancillary ligands were synthesized and characterized. The experiment results from in vitro cytotoxicity assay and molecular docking shows that the Iridium (III) complex activity is equal to standard Cisplatin which is also supported by computational docking studies. 

*Experimental*

## Materials and Methods

The analar grade of salicyaldehyde, hydrazinehydrate, distilled ethanol, thiosemicarbazide, Iridium (III) chloride trihydrate and solvents were purchased from Merck. Thin layer chromatography (TLC) was performed using TLC plates coated with silica gel. Petroleum ether and ethyl acetate was used as eluant on TLC. Spots were identified using iodine. The column chromatography technique was used to purify the crude sample using silica gel as adsorbent. IR spectra were recorded in Schimadzu (ATIR) and the absorption frequencies quoted in reciprocal centimeters. ^1^H-NMR spectra were recorded in a 400 MHz NMR Spectrometer. TGA-DSC curves were recorded on Shimadzu 60H model at 10˚C/min. heating rate. Powder X ray diffraction pattern were recorded using Bruker diffractometer model D8.

*General Procedure for the preparation of ligands HL*_1_ *– HL*_4_

**Figure 1:**
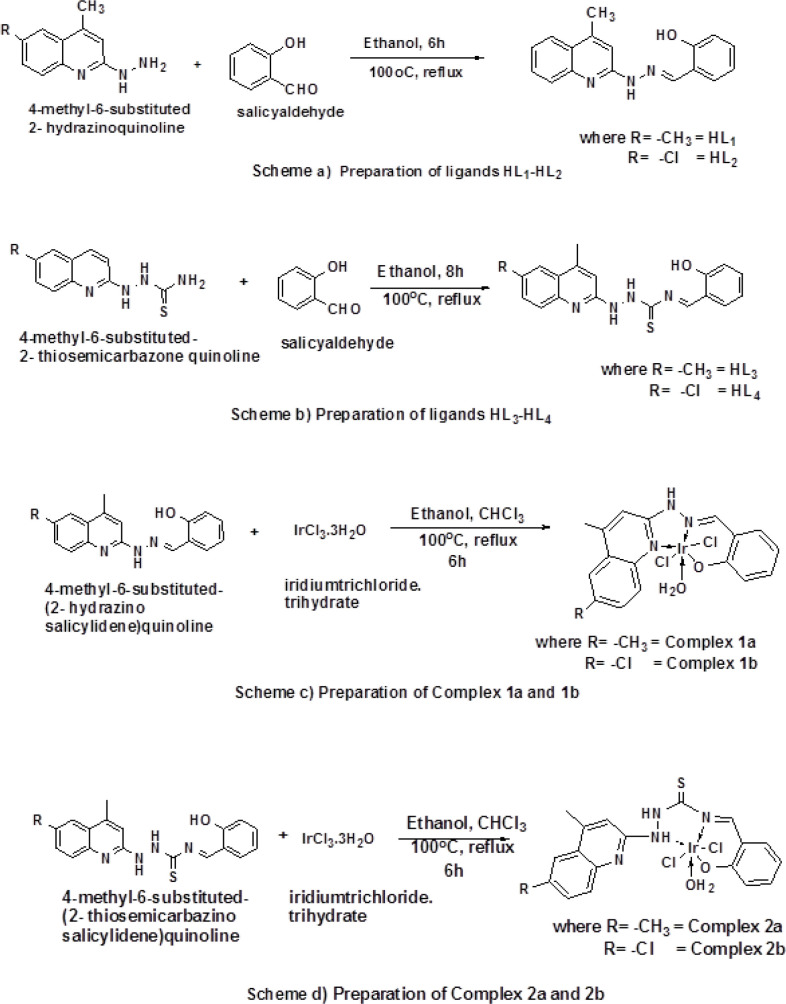
Schematic Diagram of Prepared Ir (III) Complexes: a. Preparation of ligands HL_1_-HL_2_; b. Preparation of ligands HL_3_-HL_4_; c. Preparation of complex 1a and 1b; d. Preparation of complex 2a and 2b

The ligands (HL_1_ – HL_4_) were obtained by the following steps. First step was refluxing the 2-chlorosubstituted quinoline in ethanol and hydrazine hydrate (for HL_1 _and HL_2_) and thiosemicarbazide (for HL_3_ and HL_4_) in ethanol for 24 h at 80^o^C. The corresponding hydrazones and thiosemicarbazones products obtained were dissolved in ethanol and added slowly to the magnetically stirred solution of salicyaldehyde. In the second step, the reaction mixture was stirred for 6 h and the completion of the reaction was checked by TLC. The resulting solution was concentrated to half of its volume. The product regenerated from the solution was washed with petroleum ether and it was recrystallized from ethanol (Figure 1a and 1b). 

*General Procedure for the preparation of Iridium (III) Complexes 1a,1b,2a and 2b*

A hot ethanolic solution of respective ligands was added slowly to ethanolic solution of IrCl_3_.3H_2_O (0.1g, 0.3355 mM) with continuous shaking and the colour changed from yellowish green to intense colour during addition. The resulting solution was refluxed for about 6 h and the solid mass was precipitated and filtered/washed by petroleum ether. It was then recrystallized with a mixture of chloroform and ethanol (Figure 1c and 1d).

*DNA nicking assay*

2 μL of the Ir complexes were added to 5 μL of plasmid DNA (PBR322) along with 2 μL of hydrogen peroxide and incubated for 1 hour at 37^o^C. The resulting sample was loaded in 1.2% agarose gel electrophoresis and results were recorded by using a UV transilluminator. 

*Cytotoxicity studies*

The anticancer activity of the synthesized compounds was carried out against HeLa cancer cell line by MTT assay method. The cell line was cultured and preserved in DMEM medium along with antibiotics in a T-shaped flask kept under incubator chamber at 37.5^o^C with 5% CO_2_ for 24-72 hrs. Samples and control were prepared in different concentrations (1mg/1mL), after mixing well in the cell cultural plate, cells were kept for 24 hrs in 5% CO_2_ incubator. After incubation, cells were washed with trypsin and added MTT dye in the cell cultured plate. These plates were again incubated for 24 hrs in 5% CO_2_ atmosphere. Thereafter the optical density value was monitored from ELISA reader at 570nm. % of cell death = (Control OD -Sample OD)/Control OD x 100.

*Molecular Docking*

Human epidermal growth factor receptor was downloaded from Protein Data Bank (PDB) with the following PDB ID: 2ITY. Docking analysis was performed by Autodock 4.0 tool. 3D structure of protein and ligand were prepared for docking analysis with MGL Tool 1.5.6. Grid box was prepared as X=56, Y=68, Z=54 with grid-point spacing angstrom of 0.858Å. The X, Y, Z coordinates were specified for grid point value of X= -58.166, Y= -5.006, Z= -21.212 and total grid points per map of 216315 were constructed enveloping the human epidermal growth factor receptor. The given input parameters were analyzed using a genetic algorithm and set 100 runs for each docking process. Finally, the best conformation was selected with the lowest binding energy, ligand efficiency, with more number of hydrogen bonds.

**Table 1:** IR Data of Ligands and Complexes in cm^-1^

Compound	υ (O-H)	υ (N-H)	υ (C=N)	υ (C-O)	υ (N-H)	υ (C=S)
HL_1_	3289	3050	1608	1253	-	-
HL_2_	3129	3030	1600	1270	-	-
HL_3_	3320	3126	1590	1271	2950	1362
HL_4_	3317	3263	1592	1270	3170	1366
1a	3386	3089	1565	1110	-	-
1b	3380	3150	1574	1095	-	-
2a	3350	3100	1575	1048	2998	1398
2b	3381	2979	1589	1066	2926	1390

**Figure 2:**
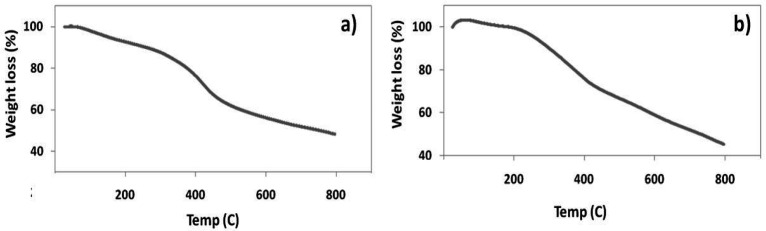
a) TGA Curve of Iridium Complex 1a, b) TGA Curve of Iridium Complex 2a

**Table 2:** ^1^H- NMR Data of Ligands and Complexes (in ppm)

Compound	δ (O-H)	δ (N-H)	δ (N-H)	δ (aromatic & HC=N)	δ (CH_3_)	δ (CH_3_)
HL_1_	9	11.2	-	7.8-7.0	3.3	2.4
HL_2_	8.8	11.3	-	7.5-6.8		1.4
HL_3_	9.7	11.4	10.3	8.3-6.9	3.3	2.3
HL_4_	9	11.3	10.8	7.8-6.8		2.3
1a	5.3	11.2	-	7.8-7.0	3.4	2.4
1b	5.8	11.3	-	7.5-6.8		1.5
2a	6.1	11.4	9.8	8.0-6.8	3.3	2.4
2b	6.3	11	9	7.9-6.8		2.5

**Figure 3:**
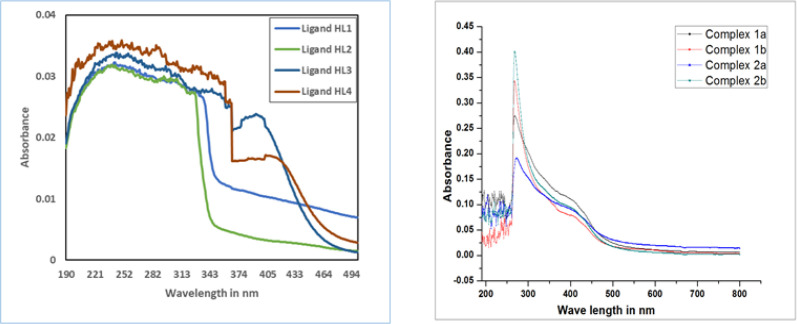
Absorption Spectra of Ligands (a) and metal complexes 1a, 1b, 2a and 2b in DMSO medium of 10^-5 ^concentrations (b)

**Figure 4:**
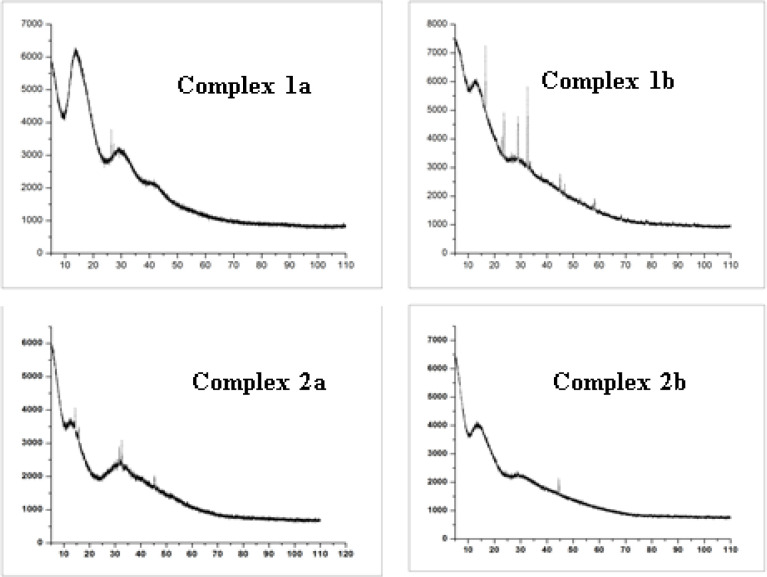
X Ray Diffractogram of Iridium Complex 1a, 1b, 2a & 2b

**Figure 5:**
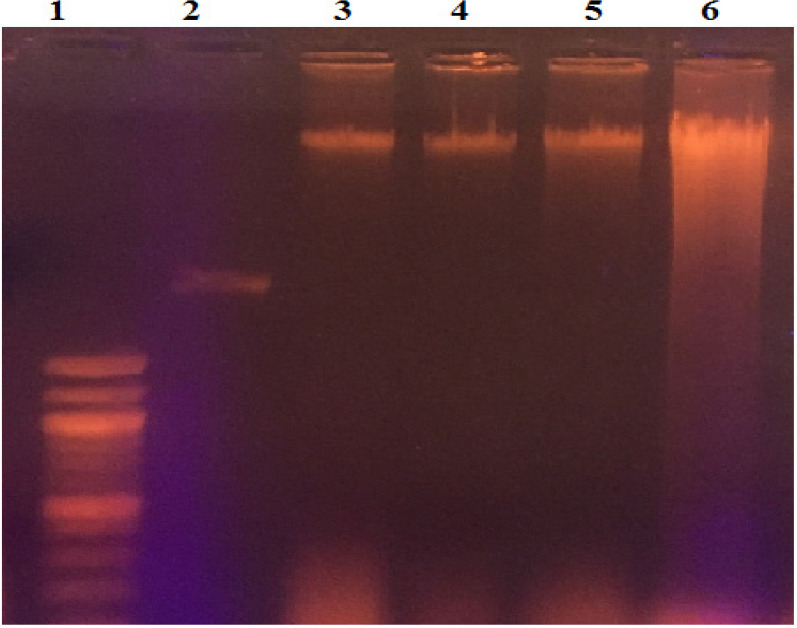
DNA Cleavage of Various Lanes of Metal Complexes - Lane 1, Marker (1kb Ladder); Lane 2, Control DNA; Lane 3, Complex 1a+ DNA; Lane 4, Complex 1b+ DNA; Lane 5, Complex 2b+ DNA; Lane 6, Complex 2a+ DNA

**Figure 6:**
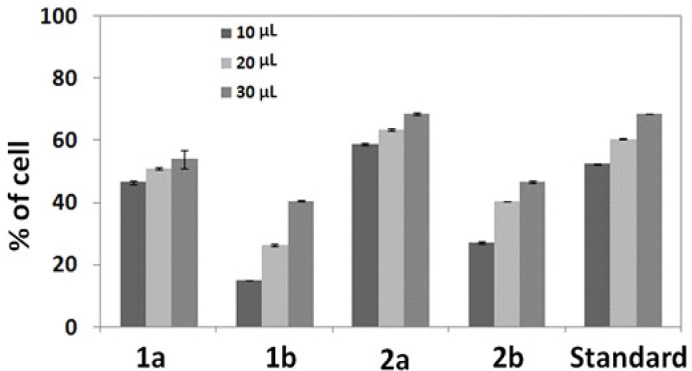
Percentage Cell Death of Metal Complexes 1a, 1b, 2a and 2b

**Table 3:** Interaction of Synthetic Compounds with Human Epidermal Growth Factor Receptor

Compounds	Binding energy (Kcal/mol)	Ligand efficiency (Kcal/mol)	Hydrogen bond interaction residues with atoms	Distance between residues (A^o^)
HL_1_	-5.26	-0.24	GLY863:H:O;	2.6
HL_3_	-5.5	-0.22	ALA864:O:H	2.1
			GLU868:OE1:HN;	2.7
			ARG889:O:H	2.4
Complex 1a	-6.17	-0.21	GLU866:OE1:H	2.2
Complex 2a	-7.04	-0.23	GLY857:O:H;	2.5
			LEU858:O:H	2.7

**Figure 7:**
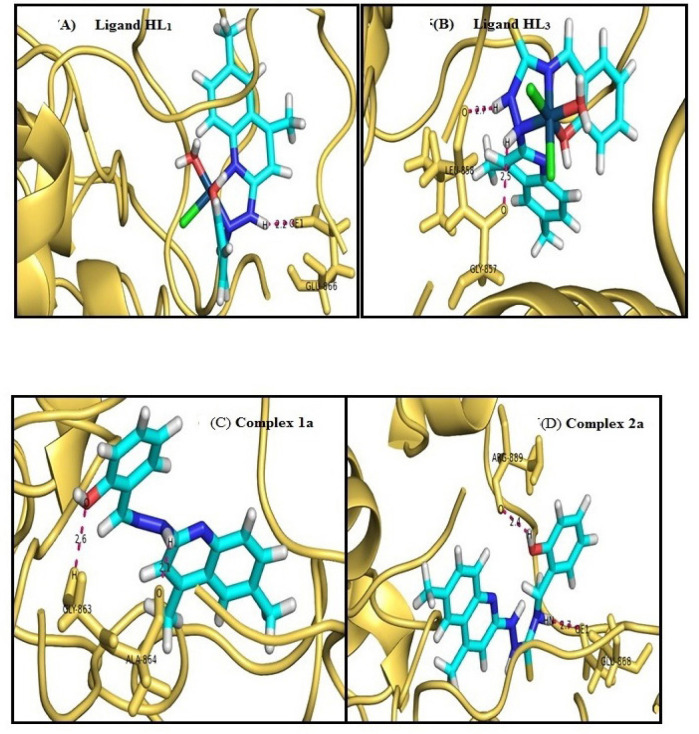
Human Epidermal Growth Factor Receptor Interacted with Ligand are Represent by Yellow Orange, Cyan and Magenta Colors Respectively. Docking pose of A) ligand HL_1_ B) ligand HL_3_ C) Complex 1a D) Complex 2a

## Results

*Ligands and Ir (III) Complexes*

A schematic diagram of synthesized ligands (HL_1_-HL_4_) and complexes (1a, 1b, 2a and 2b) is given in the Figure 1. All the ligands are soluble in common organic solvents (ethanol, chloroform, methanol and acetone). The metal complexes are partially soluble in common organic solvents and completely soluble in DMSO and DMF All the synthesized complexes were found to be stable in air and light. Molar conductivity measurements envisage the nature of the molecule to be electrolytic or non-electrolytic. The study of electrolytic behaviour using conductivity bridge (Equiptronics Conductivity Meter Model No-E660) of metal complex 1a, 1b, 2a and 2b in solutions (DMF) provides brief insight on the nature and composition. The measured molar conductance of the metal complexes 1a, 1b, 2a and 2b were found to be in the range of 25-53 Ω^-1^cm^2^ mol^-1^. It was evidenced that if conductance in DMF medium is less than 60 Ω^-1^cm^2^ mol^-1^, substance is non-electrolyte in nature and if it conductance is greater than 60 Ω^-1^cm^2^ mol^-1^, substance is electrolyte in nature. The values obtained for the complexes 1a, 1b, 2a and 2b in our study indicates that synthesized complexes are non-electrolytic in nature. (Ali et al., 2013; Wang et al., 2018).

*Spectroscopic studies*

The coordination behaviors of ligands were determined by comparing the vibrational frequency of the ligands and their metal complexes (Abd-Elzaher et al., 2016; Kumar et al., 2016; KantiSeth et al., 2015). The IR spectrum of the ligands (HL_1_- HL_4_) showed characteristic stretching frequency for -OH, -NH, -C=N and C-O groups as shown in the (Table 1) [IR Spectra Figures S1-S8 are given as supplementary information]. The –OH, –NH and –C-O groups of the ligands (HL_1_- HL_4_) showed a stretching frequency in the range of 3317-3129 cm^-1^, 3,263-2,950 cm^-1^ and 1,271-1,253 cm^-1^. The peak at the range of 1608-1590 cm^-1^ of the ligands is attributed to azomethine group. The C=S group for the ligands (HL_3_ and HL_4_) resonates at 1,362 and 1,365 cm^-1^. The IR spectrum of the metal complexes (1a, 1b, 2a and 2b) exhibits broad peaks in the region of 3,386-3,350 cm^-1^ indicating the presence of coordinated water molecules. Disappearance of sharp signal of –OH of the ligands and shift in the stretching frequency of C-O group in the metal complexes infers that the ligands are coordinated through the phenolic oxygen of the salicylaldehyde ring. The strong absorption of complex 1a, υC=N at 1,565 cm^-1^ and υN-H at 3089 cm^-1^ compared to that of the ligand HL1 reduced to lower wave numbers suggested the coordination to the metal occurs through the –NH and azomethine groups. The retaining of the peak of υC=S group in the metal complex 2a and 2b indicates that they are not participated in bonding. In conclusion, these data revealed that the ligands are coordinated with NNO donor fashion to the metal. 

The ^1^H NMR spectrum (Kanti Seth et al., 2017; Hasan et al., 2015; Sinha et al., 2015; Abu Shamma Hijazi et al., 2018) of the ligands (HL_1_-HL_4_) resonates at 6.5-7.9 ppm assigned to aromatic protons as shown in the (Table 2) (^1^H-NMR spectra Figures S9-S16 are given as supplementary information). The 4-methyl protons of the ligands viewed at 1.4 - 2.5 ppm appeared as singlet. The Peak at 11.0-11.4 ppm and 8.8-9.7 ppm was attributed to the -NH and -OH groups. The –NH group of thiosemicarbazino ligand (HL_3_ and HL_4_) resonantes at 10.3 and 10.8 ppm. The proton NMR spectral values (Table 2) of the metal complexes 1a, 1b, 2a and 2b, methyl protons signal appeared around 1.2-2.8 ppm. The disappearance of phenolic –OH peak at 8.8-9.7 ppm and appearance of signals at 5.3-6.3 ppm indicates that the presence of coordinated water molecules. The peak at δ10.3 and 10.8 ppm corresponds to NH of the ligands (HL_3_ and HL_4_) shifted to δ9.8 and 9.0 ppm indicates the ‘NH’ is coordinated to metal. Hence the ‘δ’ value shifted from δ10.3 to 9.8 and 10.8 to 9.0 compared to ligand, confirmed the mode of coordination of ligand to metal. From the spectral values it is confirmed that the ligand is coordinated through C-O, -NH and -CH=N to the metal.

*Thermal Analysis*

Thermo Gravimetric Analysis (TGA) was used to analyze the presence of water molecules outside the coordination sphere in the metal complexes, decomposition temperature and stability of the complexes(Yang et al., 2015). TGA for the complexes were analyzed (heating rate increment is 20ºC min^-1^) under nitrogen atmosphere. Weight loss was measured from ambient temperature upto 800 ºC. The TGA curve of the metal complex 1a showed different phase decomposition as shown in the Figure 2a. The initial decomposition was observed at a temperature 308ºC (17%). The calculated weight loss in percentage implies the loss of one coordinated water molecule and two chlorine atoms. The second phase decomposition observed in a temperature value of 476.1 ºC. Weight loss in percentage with a range of (40%) indicates the ligand decomposition and leaving the metal residue as iridium oxide. The TGA curve of metal complex 2a showed decomposition in three steps as shown in the Figure 2b. The first stage decomposition at a range 200ºC (3%) measures the loss of one coordinated water molecule. The second stage decomposition was obtained with the gradual decrease in the temperature range of 305 ºC. The weight loss in percentage at the range of (12%) is corresponding to the cleavage of two chlorine atoms and after that gradual decrease at 700 ºC leaves the metal residue as iridium oxide similar to complex 1a. Complex 1b and 2b has also showed the loss of one coordinated water molecule and two chlorine atoms similar with 1a & 2a complexes (data not shown).

*UV – Visible absorption spectroscopy*

The absorption spectra of the Ir (III) complexes 1a, 1b, 2a and 2b Figure 3. shows Metal-to-Ligand-Charge Transfer (MLCT) where an electron is promoted from a metal d orbital to a vacant π* orbital of the ligand (along with Ligand–Centered (LC) π-π* transitions). The synthesised compounds HL_1_- HL_4_ showed that three absorption bands for the ligands at 221-261, 282-313 and 375-403 nm (Figure 3a). The band from 221-261nm is assigned to π-π* transition of the aromatic rings, absorption at 282-313 nm is due to- n-π* transition -of C=N group. Charge transfer transition is assigned for the band at 375-403 nm (Zhou et al., 2017). The metal complexes 1a, 1b, 2a and 2b exhibited absorption bands at 325-329 nm ascribed to ^1^LLCT transitions as shown in the Figure 3a. In addition, band at 408-425 nm is contribution of spin allowed ^1^MLCT and ^1^LC transitions and low intense band above 450nm is due to spin forbidden singlet to triplet metal to ligand charge transfer and the strong spin orbit coupling effects of iridium atom of the complexes.

*Powder XRD*

Powder X-ray diffraction measurement (XRD) considered as an alternative tool to get information about the geometric and inter atomic arrangements of the molecules. The crystal lattice parameters of the complexes of iridium complexes 1a and 2a were measured on Bruker D8 XRD advanced diffractometer in the range 5º to 110º of 2 theta value Figure 4. The three 2θ peaks are corresponds to (111), (200) and (220) planes and the lattice parameters were calculated (Paula et al., 2013; Boultif et al., 2004). The lattice parameter of the complex 1a were found to be a=7.915, b=18.4558, c=14.2421, α=90.000, β = 95.025, γ =90.00 and the data revealed that the complex 1a belongs to the monoclinic crystal. The lattice parameter of the complex 2a were found to be a=7.9010, b=18.4655, c=14.3158, α= 90.000, β= 84.959, γ =90.00 and it also possess monoclinic crystal system. Similarly, the lattice parameter of the complex 1b and 2b were also indicated that the synthesized complexes are monoclinic crystal system.

*DNA Cleavage study*

Investigations on interaction of DNA with small molecules are basic in the design of new drug in the field of pharmaceutical chemistry. Some metal complexes interact with DNA and induce the breakage of DNA strands. Thus to cancer genes, after DNA strand cleaves, the DNA double strands break, the replication ability of the cancer gene is destroyed (Soundararajan et al., 2017; Ganapathy et al., 2017; Aduri et al., 2017; SurendraBabu et al., 2017; Kavitha et al., 2016). To access the DNA cleavage ability of the metal complexes 1a, 1b, 2a and 2b (Figure 5) supercoiled plasmid DNA (PBR322) along with hydrogen peroxide was incubated DNA for 1 hour at 37^o^C. Incubated samples were loaded into agarose gel electrophoresis. To the 1.2 % Agarose gel, along with DNA ladder (Biolab) all the samples were loaded separately with 50V to examine the DNA cleavage. The study revealed that the compounds has cleaved the DNA and produced the smeared DNA while running on 1.2% agarose gel.

*Cytotoxicity Studies*

Cellular level in vitro cytotoxicity study was carried out against HeLa cancer cell line for four of the metal complexes (1a, 1b, 2a and 2b) compared with the standard drug Cisplatin (Mandal et al., 2014; Yun et al., 2007). The stock solutions of the samples were prepared with the concentration of 1mg/lmL and distributed in the cells as 10 μL, 20 μL and 30μL. The comparative analysis of cytotoxic activity of compounds with Cisplatin was conducted in order to better understand their pharmacological behavior. The two leaving –Cl groups in Cisplatin are responsible for their pharmacokinetics, as same in the case of our compounds. The electronic effect on the metal coordinated ligand might influence the strength of M-C, M-O and M-N bond as well as the rate of hydrolysis which in turn affects the anticancer activity of the complex as mentioned. The compounds (1a, 1b, 2a and 2b) were analyzed, the structure of metal complexes having the chelated system (i-e) NNO fashion, which stabilize the compounds. The two –Cl groups present in the compounds leads to fast reduction and high toxicity. On comparing with the standard, the compounds 1a and 2a were more cytotoxic towards the cell lines than 1b and 2b as shown in the Figure 6.

*Molecular Docking*

Molecular docking analysis of human epidermal growth factor receptor (PDB ID: 2ITY) interacted with synthetic compounds Table 3 (ligand (HL_1_ and HL_3_) and metal complex 1a and 2a) (Morris et al., 2009; Mohammed et al., 2017; Yin et al., 2010; Ang et al., 2011]. From these analysis Figure 7A, HL_1_ atomic interaction between hydrogen, oxygen atoms of GLY863, ALA864 (-5.26Kcal/mol) and oxygen, hydrogen atoms of HL1 compound and Figure 7B, HL3 shown atomic interaction between OE1, oxygen atoms of GLU868, ARG889 (-5.5Kcal/mol) and HN, oxygen atoms of HL_3_ compound. Complex 1a shows atomic interaction between OE1 atom of GLU866 (-6.17Kcal/mol) and hydrogen atom of 1a compound and complex 2a shows atomic interaction between oxygen’s atoms of GLY857, LEU858 (-7.04 Kcal/mol) and hydrogen’s atoms of 2a compound in Figures 7C and 7D. From the results obtained the binding energy of the synthetic compounds are in the order Complex 2a < Complex 1a < HL_3_ < HL_1_ and their bond length between the binding sites falls within the limited range for all the synthetic compounds (3A^o^) in Table 3 and also the results inferred that apart from the H-O (amino acid- HL_1_, HL_3_, Complex 1a and 2a), O-H (amino acid- HL_1_, HL_3_, Complex 1a and 2a), O-NH- (aminoacid-HL_3_) are the prominent site of binding between the enzyme and the suspected drug in comparison with Complex 1b and 2b. 

In conclusion, a series of novel four iridium (III) complexes (1a, 1b, 2a and 2b) of 4,6-dimethyl-2-(salicylidenehydrazino)quinoline HL_1_, 4-methyl-6-chloro-2-(salicylidene hydrazino) quinoline HL_2_, 4,6-dimethyl-2-(salicylidenethiosemicarbazino)quinoline HL_3_ and 4-methyl-6-chloro-2-(salicylidenethiosemicarbazino) quinoline HL_4_ were synthesised. All the synthesised ligands and complexes were well characterized by spectral and thermal analysis. The complexes were found to exhibit octahedral geometry with monoclinic crystal lattice. DNA cleavage studies were analysed using plasmid DNA (PBR322) along with hydrogen peroxide by agarose gel electrophoresis. The studies explained that the complexes have the ability to cleave the supercoiled DNA. The in vitro cytotoxicity studies was investigated against HeLa Cancer Cell Line by MTT assay and the findings are the complex 1a and 2a showed better cytotoxicity almost equal to standard drug Cisplatin. Further the complexes were docked with human epidermal growth factor receptor and found to be a good interaction with compounds.
